# Developing a Novel Machine Learning-Based Classification Scheme for Predicting SPCs in Breast Cancer Survivors

**DOI:** 10.3389/fgene.2019.00848

**Published:** 2019-09-18

**Authors:** Chi-Chang Chang, Ssu-Han Chen

**Affiliations:** ^1^School of Medical Informatics, Chung Shan Medical University, Taichung, Taiwan; ^2^IT Ofﬁce, Chung Shan Medical University Hospital, Taichung, Taiwan; ^3^Department of Industrial Engineering and Management, Ming Chi University of Technology, New Taipei City, Taiwan

**Keywords:** second primary cancers (SPCs), breast cancer, machine learning, classification, machine learning-based classification scheme

## Abstract

Due to the high effectiveness of cancer screening and therapies, the diagnosis of second primary cancers (SPCs) has increased in women with breast cancer. The present study was conducted to develop a novel machine learning–based classification scheme for predicting the risk factors of SPCs in breast cancer survivors. The proposed scheme was based on the XGBoost classifier with the following four comparable strategies: transformation, resampling, clustering, and ensemble learning, to improve the training balanced accuracy. Results suggested that the best prediction accuracy for an empirical case is the XGBoost associated with the strategies of resampling and clustering. The experimental results showed that age, sequence of radiotherapy and surgery, surgical margins of the primary site, human epidermal growth factor, high-dose clinical target volume, and estrogen receptors are relatively more important risk factors associated with SPCs in patients with breast cancer. These risk factors should be monitored for the early detection of breast cancer. In conclusion, the proposed scheme can support the important influence of personality and clinical symptom representations in all phases of the primary treatment trajectory. Our results further suggested that adaptive machine learning techniques require the incorporation of significant variables for optimal predictions.

## Introduction

The effectiveness of cancer screening and therapies has resulted in an increase in the number of diagnosed second primary cancers (SPCs) throughout the world. Breast cancer is the most commonly diagnosed malignant tumor in women (Mellemkjaer et al., 2006; Kamińska et al., 2015; The Taiwan Cancer Registry, 2019a; The Taiwan Cancer Registry, 2019b). In Taiwan, breast cancer is the main type of cancer found in women. The age-adjusted incidence rates have increased from 12.07 per 100,000 women in 1979 to 73.60 per 100,000 women in 2016 (The Taiwan Cancer Registry, 2019a; The Taiwan Cancer Registry, 2019b). The five-year survival rate after breast cancer treatment has been reported to be approximately 84.97% (Huang et al., 2016). The definition of Multiple Primary Malignant Neoplasms was first published in 1932 by Warren and Gates. According to the report by Warren and Gates, both the primary and secondary tumors should be malignant with histologic confirmation, and there should be at least 2 cm of normal tissue between the two tumors. In addition, the tumors should be separated in time by at least 5 years and metastatic tumors should be excluded (Warren and Gates, 1932). In this study, we aimed to create a novel machine learning–based classification scheme for predicting the risk factors of SPCs in breast cancer survivors. Although there are several evidence-based clinical guidelines for the diagnosis and treatment of breast cancer, only a few have addressed lifelong follow-up care for breast cancer survivors. Furthermore, the treatment of breast cancer depends on the diagnostic stage, the location and size of the tumors, and tumor characteristics. Several risk factors for SPCs after treatment for breast cancer have been reported, which include environmental, smoking and alcohol use (Kolak et al., 2017), obesity (Fassier et al., 2017), cancer susceptibility genes (Yousefi et al., 2018), or previous treatments received (Markus et al., 2019)

Evidence has come from different sources, whereas, methods for synthesizing all the evidence are required. To further improve the outcomes in patients with breast cancer, physicians must identify the risk factors responsible for poor survival rates and they must develop applicable treatment strategies. Secondary cancer is due to the lack of clinical treatment strategies as well as the absence of risk factor identification to prevent its occurrence.

Many studies have been conducted using statistical methods for cancer classification and predictions (Liu et al., 2011; Khormuji and Bazrafkan, 2016; Xie et al., 2016). However, these statistical models require the establishment of formidable model assumptions during the model construction process. When these modeling assumptions are violated, it becomes difficult to achieve the desired results. Unlike models for statistical disease prediction, cancer prediction models that are based on machine learning techniques do not require powerful model assumptions and *a priori* assumptions concerning the properties of the data. They can, however, capture delicate underlying patterns and relationships contained in empirical data and they provide promising cancer prediction results (Tseng et al., 2014; Kourou et al., 2015; Tseng et al., 2017; El Houby, 2018).

Machine learning–based cancer classification models have been used in many reports in the literature to predict breast cancer recurrence (Yu et al., 2014; Ye et al., 2018; Vural et al., 2016). However, to the best of our knowledge, no reported studies have proposed machine learning–based classification schemes for SPC classifications.

## Materials and Methods

**Figure 1:**
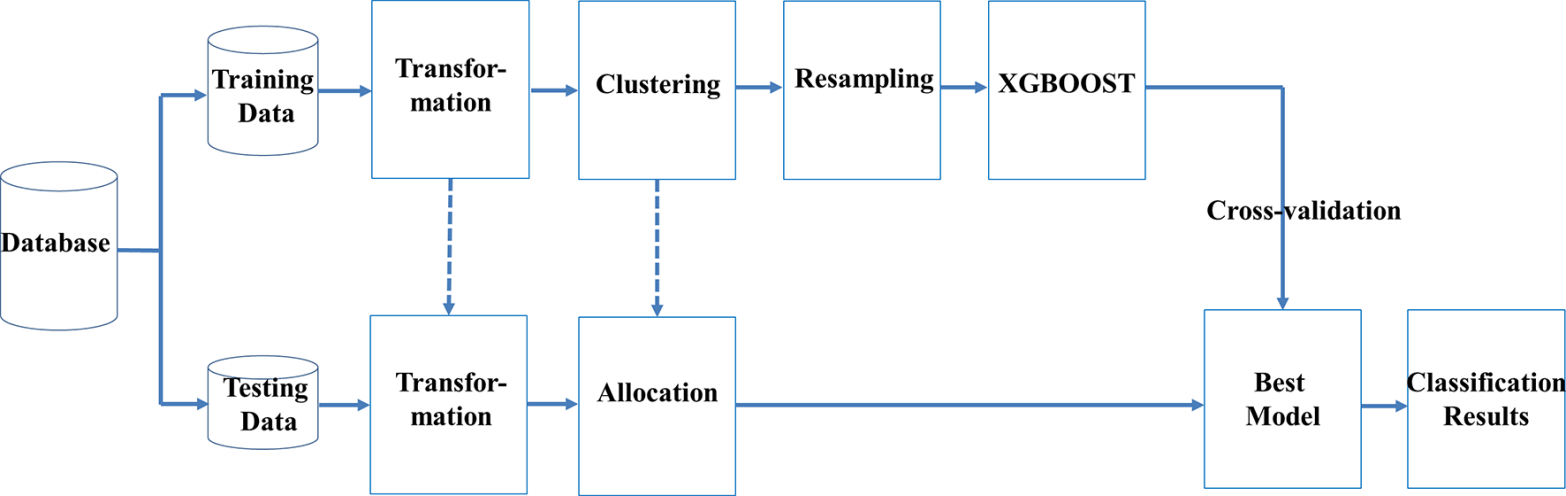
Flowchart of the proposed TCRE scheme.

We used machine learning techniques to develop a novel classification scheme, which included transformation of data, clustering, resampling, and ensemble learning (TCRE) to predict SPCs in women to have had breast cancer. In the proposed model, we first divided the original dataset into training data and testing data using specific percentages. **Figure 1** shows the flowchart for this scheme.

In the proposed TCRE scheme (**Figure 1**), the original dataset was divided into the training data and testing data with specific percentages. Both the training and testing datasets were arranged into a clear form in which the columns represent the features, and labels and rows represent the cases. Subsequently, a series of procedures were conducted using the following steps:

1. **Step 1 includes transformation to determine better feature representation.** Principal component analysis (PCA) was used to transform the original feature space into a lower dimensional space in which each dimension could be regarded as a base that explains the variability of the data best, which is similar to a noise removal process. PCA has been empirically proven in the literature to be able to improve classification results (Trivedi et al., 2015; Ikram and Cherukuri, 2016; Nasution et al., 2018). The classifier of ensemble learning, which is described below, used the gradient descent idea in an iterative manner to identify parameters with a local minimum. The classifier always disapproves the problem curse of dimensionality and, therefore, maintaining less and insignificant data may improve the convergence speed and the quality of the classification results.
2. **Step 2 includes clustering to group cases that are similar in advance.** Previous studies have indicated that performing clustering before classification may be beneficial because new grouping information is assigned in a dummy fashion relative to the original dataset (Alapati and Sindhu, 2016; Sekula et al., 2017). The k-means or k-modes algorithm has been used for clustering training data, in which the optimal clusters number is determined by internal validation measures (Chawla et al., 2004) and rank aggregation of stability (Huang, 1998).
3. **Step 3 includes resampling to alleviate the class imbalance issue.** The SPBC datasets generally have class imbalance problems because they often comprise a much higher number of breast cancer patients without SPBCs, even though only a small percentage of patients have an SPBC. When the datasets have imbalanced classes, the classifiers struggle for accuracy with imbalanced data because they are biased toward the majority class. Worse yet, the classifiers may predict everything to belong to the majority and the minority is therefore ignored to pursue a high, but pseudo, accuracy rate (Wang and Yao, 2012; Wang and Yao, 2013; Chen and Guestrin, 2016). In this study, we focus on prediction improvements through resampling method by applying SMOTE (Synthetic Minority Oversampling TEchnique) to conduct data resampling. SMOTE builds upon two methods by up-sampling the minority class and down-sampling the majority class (Fernández et al., 2018). In addition, we applied oversampling technique and try to preprocess imbalanced data before we feed them into a classifier. The main motivation behind the need to detecting the majority class and less sensitive to the minority class.
4. **Step 4 uses ensemble learning to construct an effective classifier that can classify patients diagnosed with SPCs accurately.** In step 4, the eXtreme gradient boosting (XGBoost), which was proposed by Chen and Guestrin (2016), is built based on the principles of gradient boosting trees. Trees can be efficiently constructed, and computations can be operated in parallel. The XGBoost was used in this study because it is an effective ensemble learning algorithm that can be used for various medical issues (Schmidhuber et al., 2018; Sun et al., 2018). Other reasons for choosing the XGBoost include the presence of several ordered or categorical variables in the dataset, there is no requirement for a data distribution assumption, and tree-based methods often perform well on imbalanced datasets.

**Figure 2:**
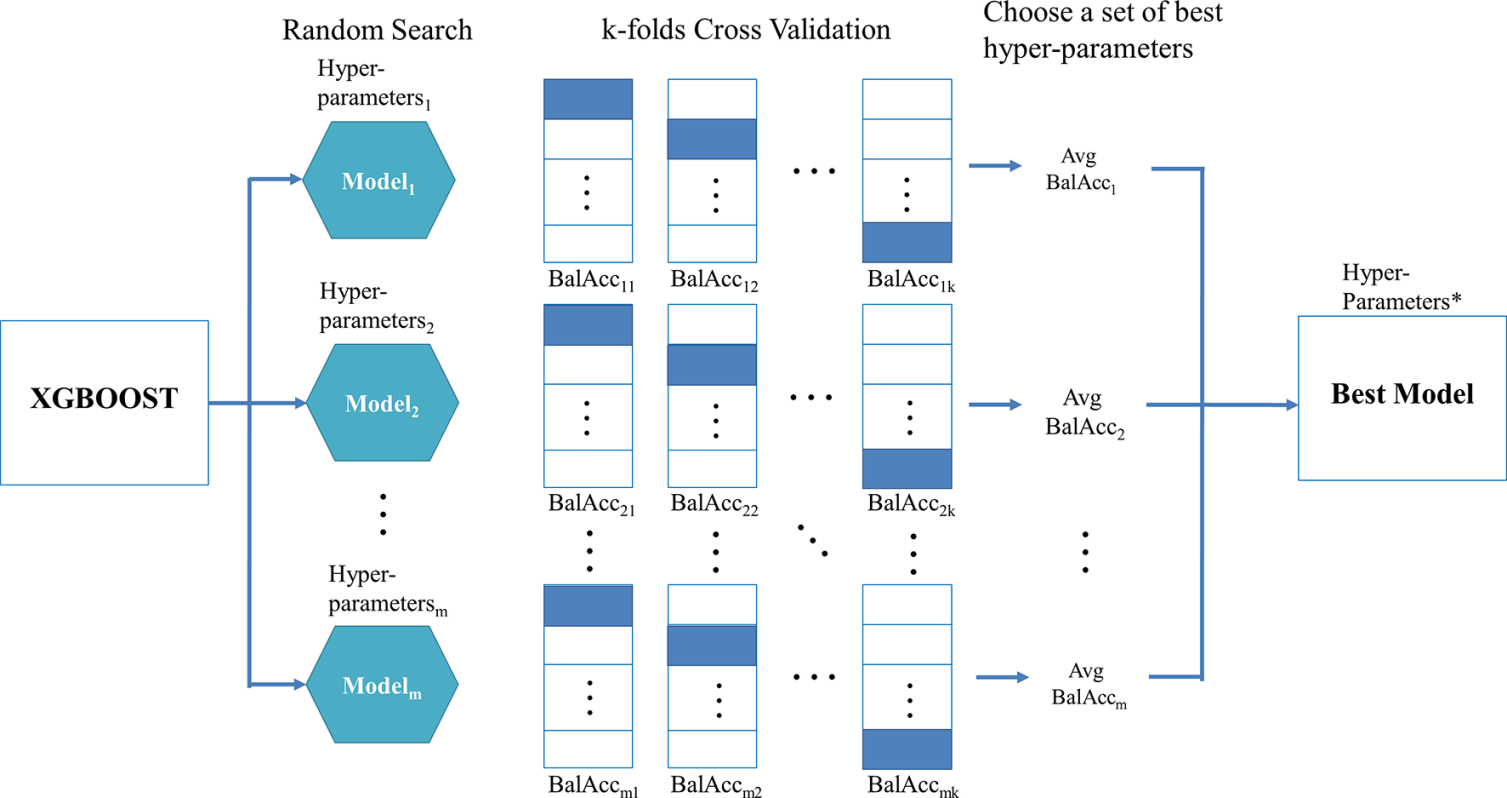
The procedure for determining the best hyperparameter set for the used XGBoost.

When using XGBoost in the proposed TCRE scheme, the primary question was how to tune the hyperparameters of this classifier during the training process to produce a model with a performance that is relatively better. As XGBoost is a flexible classifier that provides numerous hyperparameters, such as the eta, maximum depth of a tree, number of rounds for boosting, gamma, and the subsample ratio of columns (Chen and Guestrin, 2016). When constructing each tree of XGBoost, the sum of an instance weight in a child and the subsample must be minimized. However, it is nearly impossible to manually choose a good set of hyperparameter combinations. The commonly used methods to resolve this problem combine the processes of k-fold cross-validation, a random search, and metric evaluation. **Figure 2** depicts the proposed procedure for identifying the best hyperparameter set for XGBoost.

**Figure 2** shows the random search scheme that implements a randomized search over the hyperparameters for *m* times in which each value of the setting is derived from a uniform distribution over all possible values of the hyperparameters. Then, for each set of hyperparameters, the training data is randomly divided into *k* equal-sized folds. Of the *k* folds, the k-1 folds are used as the “really” training data for training the model, and the remaining single fold is considered as the validation data for validating the performance of the corresponding hyperparameters. This process is repeated *k* times, and then the corresponding evaluation metric is calculated and subsequently averaged to produce an average value for each set of hyperparameters. The metrics, such as AUC, kappa, or balanced accuracy, are suggested in the class imbalanced dataset rather than sensitivity, specificity, or accuracy because the former set of metrics simultaneously takes the performance of each class into consideration. In particular, the balanced accuracy was adopted in this study, which considered the average values of sensitivity and specificity.

**Figure 3:**
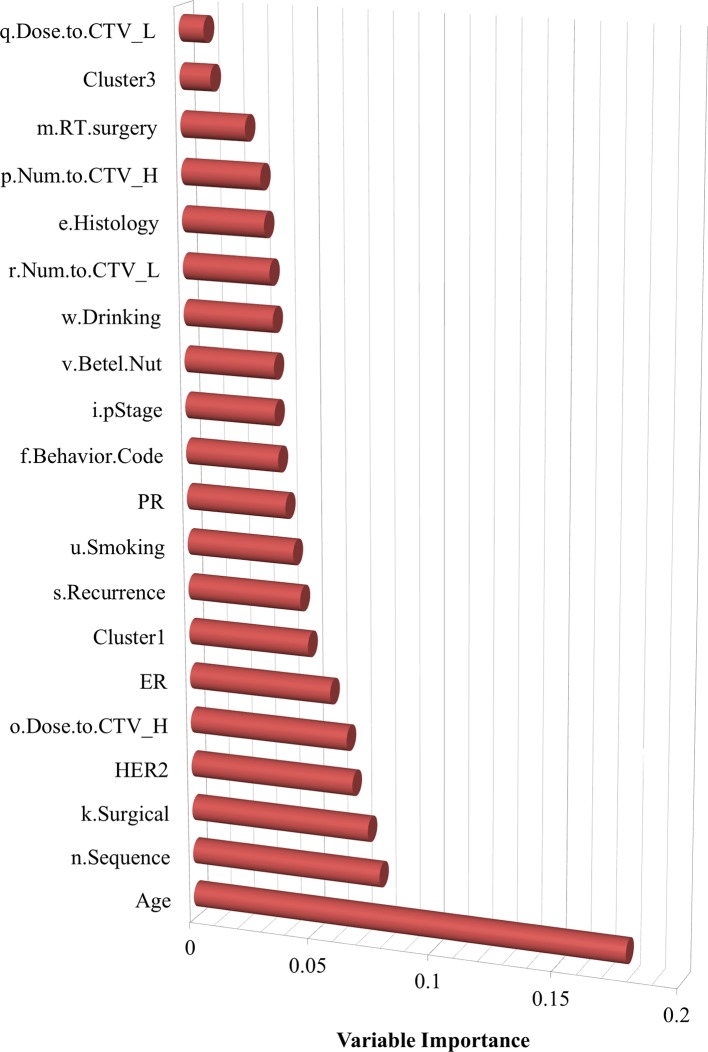
The ranked variable importance for breast cancer survivors.

In the testing phase, as shown in **Figure 3**, each sample is transformed to PCA space based on the weight matrix and the mean vector of the training data, and then the sample is allocated to its nearest clustering center. Only the features of the preprocessed testing data were fed into the best model to obtain the corresponding prediction responses. Lastly, the prediction responses were compared with the corresponding labels in the testing data to generate a confusion matrix. Furthermore, the information about variable importance was also extracted based on the training information from the best models, which was then used to identify important risk factors for breast cancer survivors.

## Results and Discussion

The medical records and the corresponding pathologic status provided by Chung Shan Medical University Hospital, Jen-Ai Hospital, and Far Eastern Memorial Hospital Tumor Registry were used for training and for evaluating the proposed methodology. The 23 predictor variables analyzed in this paper are considered to be associated with the risk factors for secondary cancer. On the basis of the comments by the expert committee and the properties of the data, the predictor variables are as follows: 1) age, 2) primary site, 3) histology, 4) behavior code, 5) differentiation, 6) tumor size, 7) pathologic stage, 8) surgical margin of the primary site, 9) surgery, 10) radiotherapy (RT), 11) RT surgery, 12) sequence of local regional therapy and systemic therapy, 13) sequence of radiotherapy and surgery, 14) dose to clinical target volume (CTV) high, 15) number to CTV high, 16) dose to CTV low, 17) number to CTV low, 18) body mass index (BMI), 19) smoking, 20) drinking, 21) human epidermal growth factor (HER2), 22) estrogen receptors (ERs), and 23) progesterone receptors.

A total of 2,964 patients diagnosed with breast cancer in three hospitals between 2010 and 2016 were included in the study. The study population involved 185 SPC cases with breast cancer. Our dataset suffered from the class imbalance problem because the total number in the class of breast cancer survivors was far less than the total number of another class of breast cancer survivors without SPCs. In addition, the dataset was randomly divided by 60% and 40% with respect to the training and testing datasets, respectively. The majority of existing studies directly use machine learning methods for cancer classification without using transformation, clustering, and resampling to deal with preprocessing and class imbalance issues (Tseng et al., 2017). Next, the built classifiers tended to predict only the majority class data, which results in a high misclassification rate of minority classes when compared with the majority class. The classification result after analysis of the data by directly using XGBoost without using transformation, clustering, and resampling (known as the single XGBoost method, which is used as a benchmark method) is shown in the first row of **Table 1**. It can be observed that the single XGBoost method provided a very high testing accuracy of >94%. However, the testing balanced accuracy was only 49.95%, which implies that all cases were classified as the majority class. Thus, the classifier single XGBoost learned nothing.

**Table 1:** Results of the sensitivity analysis and the corresponding classification results of the proposed TCRE scheme.

Transformation	Resampling	Clustering	Training accuracy	Training balanced accuracy	Testing accuracy	Testing balanced accuracy
0	0	0	0.9447	0.5104	0.9432	0.4995
0	0	1	0.9447	0.5104	0.9387	0.4971
0	1	0	0.7195	0.7604	0.6853	0.5830
0	1	1	0.7075	0.7743	0.6889	0.6000
1	0	0	0.9526	0.5803	0.9378	0.5042
1	0	1	0.9471	0.5775	0.9342	0.5023
1	1	0	0.6691	0.6629	0.6583	0.5307
1	1	1	0.6348	0.6700	0.6348	0.5638

### The Sensitivity Analysis and the Corresponding Classification Results

The proposed TCRE scheme includes the use of PCA, resampling, clustering, and XGBoost. A combination of these parameters was adopted during the training and testing processes to evaluate the performance of each preprocessing method. Such a combination implies using or not using PCA transformation, applying or not applying resampling, performing or not performing clustering before classification. However, currently, we have no idea about whether each preprocessing method was adopted in association with the model in this dataset. In addition, no specified preprocessing method combination has been confirmed to be the best; it depends on the available dataset. Therefore, a sensitivity analysis must be conducted while users are training a model. In addition, the accuracy was (TP+TN)/(P+N), where TP is a true positive, TN is a true negative, P is the number of real positive cases in the data and N is the number of real negative cases in the data. The balanced accuracy was used to deal with imbalanced datasets. The balanced accuracy is defended as (TP/P+TN/N)/2. **Table 1** presents the results of the sensitivity analysis and the corresponding classification results of the proposed TCRE scheme. **Supplementary Materials** show the detailed information as determined by the testing balanced accuracy and validation balanced accuracy within the proposed TCRE scheme.

In the first three columns of **Table 1**, the numeral 1 represents the corresponding preprocessing methods that are used, whereas, the zeros represent the methods that are not activated in this study. In the subsequent columns, the accuracy was calculated as the proportion of cases that were correctly classified, while the balanced accuracy was defined as the average value of the proportion correct in each individual class. The term training or testing represents the abovementioned metrics generated during either the training stage or the testing stage. We lastly aimed to maximize the training balanced accuracy based on the analysis. The process suggests that both resampling and clustering techniques must be adopted in the subsequent testing stage. On the basis of the optimal model selected throughout the training process described above, the rate of SPCs in women with breast cancer in the testing data was also approximately 6.2%. As shown in **Table 1**, our results show that the best scheme was the XGBoost associated with the strategies of resampling and clustering. In addition, the performance of the testing balanced accuracy and training balanced accuracy were increased by 10.05% and 26.39%, compared to previous the baseline XGBoost.

### Identification of Important Risk Factors

Variable importance was also assessed, as shown in **Figure 3**, which indicates the features that are more influential on patients with SPBC. It determined that age, the sequence of radiotherapy and surgery, surgical margins of the primary site, HER2, dose to CTV high, and ER are relatively more important risk factors associated with SPCs.

## Conclusions

On the basis of the evidence obtained in this study, it can be concluded that the positive correlation between breast cancer and SPCs is not an accidental result. Breast cancer is the most common female cancer throughout the world. Although studies of breast cancer survivors dominate the survivor literature, few prospective randomized controlled trials have intervened in breast cancer survivors. We urge caution regarding the prevention and treatment of breast cancer survivors. Risks of long-term and late-stage effects after breast cancer treatment are associated with several factors. The results of this study suggest that age, sequence of radiotherapy and surgery, surgical margins of the primary site, HER2, dose to CTV high, and ER, when appropriate, should be recommended for patients with breast cancer. Radiation, chemotherapy, and hormone/endocrine therapy with aromatase inhibitors are especially associated with an increased risk of developing SPCs in patients with breast cancer. There is sufficient evidence indicating that obesity is a risk factor for SPC development and other problems. To determine whether women with breast cancer are genetically susceptible or at high risk of developing SPCs that may affect other family members, it is necessary to collect their detailed medical history, including key risk factors, and the family history of their parents. Long-term follow-up of patients with breast cancer is important for documenting the risks and patterns of SPCs, and knowledge about these aspects will influence surveillance and prevention strategies in the future.

## Data Availability

The datasets generated for this study are available on request to the corresponding author.

## Author Contributions

C-CC initially drafted the manuscript and collected the features, analyzed the experiments and revised the paper. S-HC did part of the codes work and the experiments. All authors designed the work, read and approved the ﬁnal manuscript and are agree to be accountable for all aspects of the work.

## Funding

This work was supported by the MOST 106-2218-E-040 -001 -MY2.

## Conflict of Interest Statement

The authors declare that the research was conducted in the absence of any commercial or financial relationships that could be construed as a potential conflict of interest.
