# Supplementary material for: Developing a Novel Machine Learning-Based Classification Scheme for Predicting SPCs in Breast Cancer Survivors
Source: Front Genet. 2019 Sep 18;10:848. doi: 10.3389/fgene.2019.00848 (PMC6759630; doi:10.3389/fgene.2019.00848)
Supplement: Supplementary file 1 [file DataSheet_1.pdf]

Developing a Novel Machine Learning-based Classification Scheme for Predicting SPCs in Breast Cancer Survivors

Chi-Chang Chang[1,2,*], Ssu-Han Chen[3,*]

[1]School of Medical Informatics, Chung Shan Medical University, Taichung, Taiwan,

[2]IT Office, Chung Shan Medical University Hospital, Taichung, Taiwan

[3]Department of Industrial Engineering and Management, Ming Chi University of Technology, New Taipei, Taiwan

* **Correspondences:**

Ssu-Han Chen

ssuhanchen@mail.mcut.edu.tw (S.-H.C.)

Chi-Chang Chang

threec@csmu.edu.tw (C.-C.C.)

Tel.: +886-4-2473-0022 (ext.12218) (C.-C.C.)

Keywords: Second Primary Cancers (SPCs), Breast Cancer, Machine Learning, Classification, Machine learning-based classification scheme

Supplementary Materials

Supplementary materials show each strategy as determined by the testing balanced accuracy and validation balanced accuracy within the proposed TCRE scheme. In the first three columns of Table S1 to Table S8, the numeral 1 represents the corresponding preprocessing methods that are used, whereas the zeros represent the methods that are not activated in this study.

Table S1. Results of the testing balanced accuracy and validation balanced accuracy of the baseline strategy (Transformation-0, Resampling-0, Clustering-0)

Transformation	Resampling	Clustering	Training accuracy	Training balanced accuracy	Testing accuracy	Testing balanced accuracy
0	0	0	0.9447	0.5104	0.9432	0.4995

Confusion matrix for training data				Confusion matrix for testing data			
		Reference				Reference	
		Non-SPBC	SPBC			Non-SPBC	SPBC
Prediction	Non-SPBC	1571	91	Prediction	Non-SPBC	1046	62
	SPBC	1	2		SPBC	1	0

Statistics for training data	Statistics for testing data
Accuracy: 0.9447	Accuracy: 0.9432
Sensitivity: 0.9994	Sensitivity: 0.9990
Specificity: 0.0215	Specificity: 0.0000
Balanced accuracy: 0.5104	Balanced accuracy: 0.4995

Table S2. Results of the testing balanced accuracy and validation balanced accuracy of the Clustering strategy (Transformation-0, Resampling-0, Clustering-1)

Transformation	Resampling	Clustering	Training accuracy	Training balanced accuracy	Testing accuracy	Testing balanced accuracy
0	0	1	0.9447	0.5104	0.9387	0.4971

Confusion matrix for training data				Confusion matrix for testing data			
		Reference				Reference	
		Non-SPBC	SPBC			Non-SPBC	SPBC
Prediction	Non-SPBC	1571	91	Prediction	Non-SPBC	1041	62
	SPBC	1	2		SPBC	6	0

Statistics for training data	Statistics for testing data
Accuracy: 0.9447	Accuracy: 0.9387
Sensitivity: 0.9994	Sensitivity: 0.9943
Specificity: 0.0215	Specificity: 0.0000
Balanced accuracy: 0.5104	Balanced accuracy: 0.4971

Table S3. Results of the testing balanced accuracy and validation balanced accuracy of the Resampling strategy (Transformation-0, Resampling-1, Clustering-0)

<table>
<tr><td>Transformation</td><td>Resampling</td><td>Clustering</td><td>Training accuracy</td><td>Training balanced accuracy</td><td>Testing accuracy</td><td>Testing balanced accuracy</td></tr>
<tr><td>0</td><td>1</td><td>0</td><td>0.7195</td><td>0.7604</td><td>0.6853</td><td>0.5830</td></tr>
</table>

<table>
<tr><td colspan="4">Confusion matrix for training data</td><td colspan="4">Confusion matrix for testing data</td></tr>
<tr><td colspan="2" rowspan="2"></td><td colspan="2">Reference</td><td colspan="2" rowspan="2"></td><td colspan="2">Reference</td></tr>
<tr><td>Non-SPBC</td><td>SPBC</td><td>Non-SPBC</td><td>SPBC</td></tr>
<tr><td rowspan="2">Prediction</td><td>Non-SPBC</td><td>1123</td><td>18</td><td rowspan="2">Prediction</td><td>Non-SPBC</td><td>731</td><td>33</td></tr>
<tr><td>SPBC</td><td>449</td><td>75</td><td>SPBC</td><td>316</td><td>29</td></tr>
<tr><td colspan="4">Statistics for training data</td><td colspan="4">Statistics for testing data</td></tr>
<tr><td colspan="4">Accuracy: 0.7195</td><td colspan="4">Accuracy: 0.6853</td></tr>
<tr><td colspan="4">Sensitivity: 0.7144</td><td colspan="4">Sensitivity: 0.6982</td></tr>
<tr><td colspan="4">Specificity: 0.8065</td><td colspan="4">Specificity: 0.4677</td></tr>
<tr><td colspan="4">Balanced accuracy: 0.7604</td><td colspan="4">Balanced accuracy: 0.5830</td></tr>
</table>

Table S4. Results of the testing balanced accuracy and validation balanced accuracy of the Resampling-Clustering strategy (Transformation-0, Resampling-1, Clustering-1)

<table>
<tr><td>Transformation</td><td>Resampling</td><td>Clustering</td><td>Training accuracy</td><td>Training balanced accuracy</td><td>Testing accuracy</td><td>Testing balanced accuracy</td></tr>
<tr><td>0</td><td>1</td><td>1</td><td>0.7075</td><td>0.7743</td><td>0.6889</td><td>0.6000</td></tr>
</table>

<table>
<tr><td colspan="4">Confusion matrix for training data</td><td colspan="4">Confusion matrix for testing data</td></tr>
<tr><td colspan="2" rowspan="2"></td><td colspan="2">Reference</td><td colspan="2" rowspan="2"></td><td colspan="2">Reference</td></tr>
<tr><td>Non-SPBC</td><td>SPBC</td><td>Non-SPBC</td><td>SPBC</td></tr>
<tr><td rowspan="2">Prediction</td><td>Non-SPBC</td><td>1099</td><td>14</td><td rowspan="2">Prediction</td><td>Non-SPBC</td><td>733</td><td>31</td></tr>
<tr><td>SPBC</td><td>473</td><td>79</td><td>SPBC</td><td>314</td><td>31</td></tr>
<tr><td colspan="4">Statistics for training data</td><td colspan="4">Statistics for testing data</td></tr>
<tr><td colspan="4">Accuracy: 0.7075</td><td colspan="4">Accuracy: 0.6889</td></tr>
<tr><td colspan="4">Sensitivity: 0.6991</td><td colspan="4">Sensitivity: 0.7001</td></tr>
<tr><td colspan="4">Specificity: 0.8495</td><td colspan="4">Specificity: 0.5000</td></tr>
<tr><td colspan="4">Balanced accuracy: 0.7743</td><td colspan="4">Balanced accuracy: 0.6000</td></tr>
</table>

Table S5. Results of the testing balanced accuracy and validation balanced accuracy of the Transformation strategy (Transformation-1, Resampling-0, Clustering-0)

Transformation	Resampling	Clustering	Training accuracy	Training balanced accuracy	Testing accuracy	Testing balanced accuracy
1	0	0	0.9526	0.5803	0.9378	0.5042

Confusion matrix for training data				Confusion matrix for testing data			
		Reference				Reference	
		Non-SPBC	SPBC			Non-SPBC	SPBC
Prediction	Non-SPBC	1571	78	Prediction	Non-SPBC	1039	61
	SPBC	1	15		SPBC	8	1

Statistics for training data	Statistics for testing data
Accuracy: 0.9526	Accuracy: 0.9378
Sensitivity: 0.9994	Sensitivity: 0.9924
Specificity: 0.1613	Specificity: 0.0161
Balanced accuracy: 0.5803	Balanced accuracy: 0.5042

Table S6. Results of the testing balanced accuracy and validation balanced accuracy of the Transformation-Clustering strategy (Transformation-1, Resampling-0, Clustering-1)

Transformation	Resampling	Clustering	Training accuracy	Training balanced accuracy	Testing accuracy	Testing balanced accuracy
1	0	1	0.9471	0.5775	0.9342	0.5023

Confusion matrix for training data				Confusion matrix for testing data			
		Reference				Reference	
		Non-SPBC	SPBC			Non-SPBC	SPBC
Prediction	Non-SPBC	1562	78	Prediction	Non-SPBC	1035	61
	SPBC	10	15		SPBC	12	1

Statistics for training data	Statistics for testing data
Accuracy: 0.9471	Accuracy: 0.9342
Sensitivity: 0.9936	Sensitivity: 0.9885
Specificity: 0.1613	Specificity: 0.0161
Balanced accuracy: 0.5775	Balanced accuracy: 0.5023

Table S7. Results of the testing balanced accuracy and validation balanced accuracy of the Transformation-Resampling strategy (Transformation-1, Resampling-1, Clustering-0)

Transformation	Resampling	Clustering	Training accuracy	Training balanced accuracy	Testing accuracy	Testing balanced accuracy
1	1	0	0.6691	0.6629	0.6583	0.5307

Confusion matrix for training data				Confusion matrix for testing data			
		Reference				Reference	
		Non-SPBC	SPBC			Non-SPBC	SPBC
Prediction	Non-SPBC	1053	32	Prediction	Non-SPBC	706	38
	SPBC	519	61		SPBC	341	24
Statistics for training data				Statistics for testing data			
Accuracy: 0.6691				Accuracy: 0.6583			
Sensitivity: 0.6698				Sensitivity: 0.6743			
Specificity: 0.6559				Specificity: 0.3871			
Balanced accuracy: 0.6629				Balanced accuracy: 0.5307			

Table S8. Results of the testing balanced accuracy and validation balanced accuracy of the Transformation-Resampling-Clustering strategy (Transformation-1, Resampling-1, Clustering-1)

Transformation	Resampling	Clustering	Training accuracy	Training balanced accuracy	Testing accuracy	Testing balanced accuracy
1	1	1	0.6348	0.6700	0.6348	0.5638

Confusion matrix for training data				Confusion matrix for testing data			
		Reference				Reference	
		Non-SPBC	SPBC			Non-SPBC	SPBC
Prediction	Non-SPBC	1	1	1	0.6348	0.6700	0.6348
	SPBC	1	1	1	0.6348	0.6700	0.6348
Statistics for training data				Statistics for testing data			
Accuracy: 0.6348				Accuracy: 0.6348			
Sensitivity: 0.6304				Sensitivity: 0.6437			
Specificity: 0.7097				Specificity: 0.4839			
Balanced accuracy: 0.6700				Balanced accuracy: 0.5638			
